# Radiation-Detoxified Form of Endotoxin Effectively Activates Th_1_ Responses and Attenuates Ragweed-Induced Th_2_-Type Airway Inflammation in Mice

**DOI:** 10.3390/ijms25031581

**Published:** 2024-01-27

**Authors:** Attila Bácsi, Beatrix Ágics, Kitti Pázmándi, Béla Kocsis, Viktor Sándor, Lóránd Bertók, Geza Bruckner, Sándor Sipka

**Affiliations:** 1 Department of Immunology, Faculty of Medicine, University of Debrecen, H-4032 Debrecen, Hungary; etele@med.unideb.hu (A.B.); agics.beatrix@med.unideb.hu (B.Á.); pazmandi.kitti@med.unideb.hu (K.P.); 2 Doctoral School of Molecular Cell and Immune Biology, University of Debrecen, H-4032 Debrecen, Hungary; 3 Department of Medical Microbiology and Immunology, Faculty of Medicine, University of Pécs, H-7624 Pécs, Hungary; kocsis.bela@pte.hu; 4 Institute of Bioanalysis, Medical School and Szentágothai Research Center, University of Pécs, H-7624 Pécs, Hungary; sandor.viktor@pte.hu; 5 National Research Directorate for Radiobiology and Radiohygiene, National Public Health Center, H-1221 Budapest, Hungary; 6 Department of Athletic Training and Clinical Nutrition, University of Kentucky, Lexington, KY 40536, USA; gbruckn@uky.edu; 7 Division of Clinical Immunology, Faculty of Medicine, University of Debrecen, H-4032 Debrecen, Hungary

**Keywords:** endotoxin, lipopolysaccharide, radiation-detoxified, Th_2_-type airway inflammation, allergy, dendritic cells, ragweed, mice

## Abstract

Urbanization with reduced microbial exposure is associated with an increased burden of asthma and atopic symptoms. Conversely, environmental exposure to endotoxins in childhood can protect against the development of allergies. Our study aimed to investigate whether the renaturation of the indoor environment with aerosolized radiation-detoxified lipopolysaccharide (RD-LPS) has a preventative effect against the development of ragweed-induced Th_2_-type airway inflammation. To explore this, cages of six-week-old BALB/c mice were treated daily with aerosolized native LPS (N-LPS) or RD-LPS. After a 10-week treatment period, mice were sensitized and challenged with ragweed pollen extract, and inflammatory cell infiltration into the airways was observed. As dendritic cells (DCs) play a crucial role in the polarization of T-cell responses, in our in vitro experiments, the effects of N-LPS and RD-LPS were compared on human monocyte-derived DCs (moDCs). Mice in RD-LPS-rich milieu developed significantly less allergic airway inflammation than mice in N-LPS-rich or common environments. The results of our in vitro experiments demonstrate that RD-LPS-exposed moDCs have a higher Th_1_-polarizing capacity than moDCs exposed to N-LPS. Consequently, we suppose that the aerosolized, non-toxic RD-LPS applied in early life for the renaturation of urban indoors may be suitable for the prevention of Th_2_-mediated allergies in childhood.

## 1. Introduction

The prevalence of allergies and asthma has increased considerably over the last few decades in developed countries [1]. This phenomenon might be related to a decreasing incidence of environmental exposure to microbial agents in early childhood due to the Western lifestyle. This is supported by earlier [2,3,4,5] and more recent epidemiological studies [6,7] reporting that children growing up on a traditional farm, which affords a wide range of microbial exposures, have a reduced risk of the development of atopic disorders. Although the exact mechanism of this observed protective farm effect is not fully understood, it might be attributed to continuous exposure to the high ambient endotoxin in a farming environment [8], which influences the regulation of the innate immunity and favors the induction of Th_1_ and regulatory T-cells [9]. Endotoxins, such as lipopolysaccharide (LPS), serve as the major and essential structural component of the outer membrane of Gram-negative bacteria. The biosynthesis of and the dynamic compositional changes in LPS are tightly regulated to protect the bacteria from environmental chemical and physical stresses and help them to adapt to these challenges [10,11]. In addition to being a vital component for Gram-negative bacteria, LPS is also a major immune-stimulating part of these microbes. The recognition of LPS by the host’s immune system is a fundamental step in identifying harmful bacteria [12]. Endotoxins are present ubiquitously in the environment and can directly influence the outcome of immune responses and the susceptibility to various diseases [13].

The protective effect of a rural lifestyle can be largely attributed to interactions with the endotoxin-rich livestock environment, contact with fodder and certain dietary factors, such as the consumption of unprocessed raw cow’s milk. Indeed, increased endotoxin concentrations were found in the house dust of farmhouses compared with urban dwellings [14]. Furthermore, higher concentrations of endotoxins were also detected in the cooled and cold-stored whole raw farm milk than in the processed shop milk samples [15]. The protective effect of higher concentrations of environmental endotoxins on allergic sensitization was also demonstrated in nonfarming households [16,17]. It was found that the airway mucosa is possibly the key site for this phenomenon, as chronic pre-exposure to endotoxins protects mice against experimental asthma through the epithelial production of A20, a ubiquitin-modifying enzyme [18]. In addition, a polymorphism in the gene encoding A20 was found to be associated with allergy and asthma risk in children growing up on farms [18].

It is worth noting that a mounting body of evidence indicates that environmental exposure to endotoxins has an ambiguous Jekyll-and-Hyde relationship with allergy and asthma (reviewed in [19,20,21]). While chronic exposure to endotoxins may account for the lower risk of atopy, it has also been demonstrated that acute inhalation of a high dose of endotoxins can cause wheezing and coughing in the first year of life [22] and exacerbates pre-existing asthma in individuals already sensitized against inhaled allergens [23,24]. Cross-sectional studies, particularly those of children raised in rural European communities, have suggested that only early endotoxin exposure can protect against the development of allergic sensitization and atopic asthma [25]. These conclusions based on children living in rural or urban environments have been also supported by mouse models [26,27,28]. Thus, the effect of endotoxin exposure in allergic diseases appears to be influenced by the timing of exposure.

The impact of environmental factors on atopy is noteworthy even during fetal development. Maternal environmental effects, including maternal infections, microbiota, diet, medications, and exposure to pollutants, are crucial for immunomodulation and are usually accompanied by epigenetic changes [29]. These alterations may play a role in both the risk and protective factors associated with allergic diseases. Nevertheless, it is certain that DNA methylation and histone modifications can enhance Th_2_ responses, maintain the population of memory Th_2_ cells, or reduce the differentiation of regulatory T-cells [29]. After birth, the development of a stable microbiome and the symbiotic relationship with a diverse array of beneficial microbes in the skin, gut, and lungs are also crucial for maintaining healthy immune homeostasis and ensuring adequate immune regulation to reduce the incidence of allergic diseases. [30,31]. Intriguingly, exposure to environmental bacteria in the postnatal period may also be associated with epigenetic modifications, serving as an underlying mechanism in the protective effect against asthma development. Indeed, intranasal administration of asthma-protective bacteria in a mouse model of asthma has been shown to trigger a local pro-inflammatory response in the airways, characterized by a sustained systemic increase in IL-6. This is followed by an epigenetic modification of CD4+ T-cells leading to IL-10 induction [32]. These changes were also associated with alteration in the microbiome composition of mice, which may have played a role in protection against asthma [32]. In addition, gene expression analysis in peripheral blood mononuclear cells derived from allergic asthmatic and healthy school-age children showed that the expression of an anti-inflammatory gene, *dual-specificity phosphatase-1* (*DUSP1*) and its specific H4 acetylation, which is associated with the anti-inflammatory effects of asthma-therapeutic glucocorticosteroids, was significantly lower in asthmatic children than in healthy ones [33].

While the exposure to environmental microbes can help to establish the proper immune status, Western lifestyles lead to dysbiosis, disrupting immune tolerance and triggering the development of inflammatory diseases such as asthma and other allergic conditions [34]. Recently, a comprehensive summarizing article was published illustrating the difference in the structure of the airway and gut microbiotas between urban and rural infants. The authors suggested that urbanization-related changes in the microbiota may elevate the risk of asthma and atopic traits, likely through cross-talk with the developing immune system [35]. Together, these observations raise the possibility that renaturation of the inner urban environment for infants seems to be a plausible solution to prevent the development of allergic diseases. However, mimicking the farm effect via the restoration of endotoxin levels in the urban environment is only possible by using a tolerable dose and a non-toxic, safe form of endotoxins. The use of endotoxins and their purified derivative LPS as immunomodulating agents is severely limited by their ability to induce clinical symptoms, including shock, fever, shaking chills, bronchoconstriction and changes in the level of nonspecific bronchial hyperresponsiveness [36]. Therefore, numerous attempts using physical, chemical or biosynthetic engineering approaches have been made to attenuate LPS and to separate endotoxic and immunostimulatory activities [37,38,39,40,41]. Moreover, ionizing radiation (^60^Co-gamma) has also been used for the detoxification of LPS [42]. Radiation not only results in chemical changes in the complex molecule of native LPS (N-LPS) but also alters its biological activity. Radiation-detoxified lipopolysaccharide (RD-LPS) preparations showed decreased toxicity, while retaining their beneficial immunomodulatory effects (reviewed in [43]).

In this study, we tested the hypothesis that the non-toxic RD-LPS in aerosol form can attenuate Th_2_-type airway inflammation and can be used as a prophylactic agent in allergic diseases. Therefore, we compared the effects of RD-LPS and its native, parental form (N-LPS) in a murine model of pollen-induced allergic airway inflammation and in a human monocyte-derived dendritic cell (moDC) culture. Our results demonstrate significant preventative effects of RD-LPS vs. N-LPS in the form of an aerosol used in the ragweed-induced bronchial inflammation of mice when the aerosol was applied daily for 70 days in the environment of the animals. Furthermore, our in vitro data show that human moDCs primed with RD-LPS have a greater ability to induce T-cell polarization towards Th_1_, which helps to establish Th_1_ dominance over Th_2_. Due to its favorable immunomodulatory properties, the non-toxic RD-LPS could be a potential new tool for the prevention of airway allergies.

## 2. Results

### 2.1. Long-Term Environmental Exposure to RD-LPS Mitigates Pollen-Induced Eosinophilic Airway Inflammation in Mice

**Figure 1:**
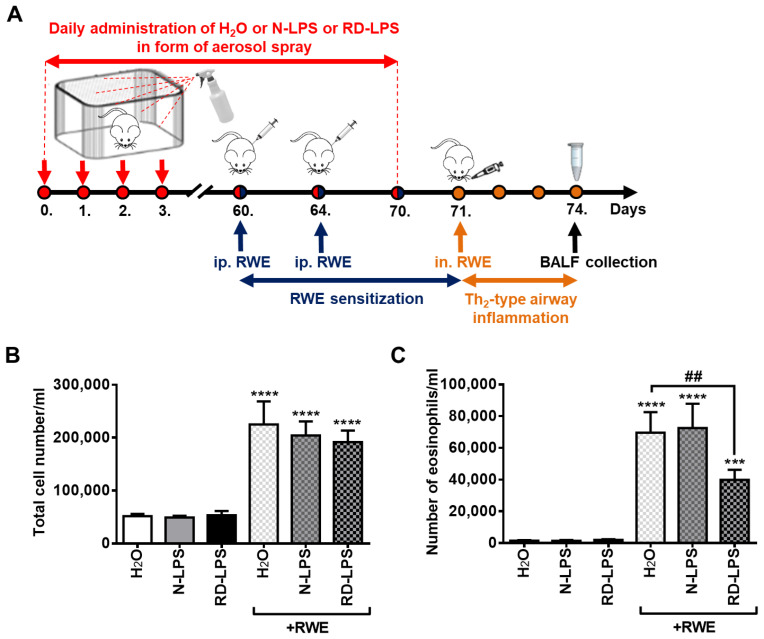
Radiation-detoxified lipopolysaccharide (RD-LPS) reduced the ragweed pollen-induced Th_2_-type airway inflammation in mice. Cages (feed and bedding) of six-week-old female BALB/c mice were treated daily for 70 days with 5 µg of native LPS (N-LPS) or RD-LPS and endotoxin-free water (H_2_O) as a control in the form of aerosol spray. To induce Th_2_-type airway inflammation, mice were sensitized intraperitoneally with endotoxin-free ragweed pollen extract (RWE), and then the allergen challenges were performed intranasally as described in the Materials and Methods section. After the euthanasia of mice, the bronchoalveolar lavage fluid (BALF) was collected, and the numbers of the infiltrating inflammatory cells were analyzed. (**A**) Schematic representation of the experimental procedure is shown. (**B**) Total cell count including eosinophils, neutrophils, lymphocytes, macrophages and (**C**) number of eosinophils were determined, separately on Wright–Giemsa-stained slides using light microscopy. For differential cell count analysis, at least 400 cells were counted. Data are presented as means ± SD of 4–10 individual experiments. *** *p* < 0.001, **** *p* < 0.0001 vs. without RWE, ## *p* < 0.01 vs. H_2_O + RWE.

To compare the in vivo immunomodulatory effects of environmental N-LPS and RD-LPS, a murine model of allergic airway inflammation was used as described in the Materials and Methods (Figure 1A). In mice kept in a mock-treated (endotoxin-free H_2_O) environment, sensitization and intranasal challenge with ragweed pollen extract (RWE) induced a significant increase in the total cell count (Figure 1B) and eosinophil cell count (Figure 1C) in the bronchoalveolar lavage fluid (BALF). Long-term (70 days) environmental exposure to either N-LPS or RD-LPS resulted in a slight decrease in the number of total cells in BALF after the intranasal RWE challenge of sensitized animals, but these changes were statistically insignificant (Figure 1B). However, prolonged environmental exposure to RD-LPS, but not N-LPS, significantly reduced the number of eosinophils in BALF (Figure 1C), which are the main inflammatory cellular components of allergic reactions and show the best correlation with pulmonary functions and Th_2_-type airway inflammation [44]. Thus, contaminating the environment with the radiation-detoxified form of LPS for 70 days may have a protective effect against ragweed pollen allergy in mice.

### 2.2. RD-LPS-Exposed Human moDCs Are More Potent Th_1_ Inducers Than the N-LPS-Treated Ones

**Figure 2:**
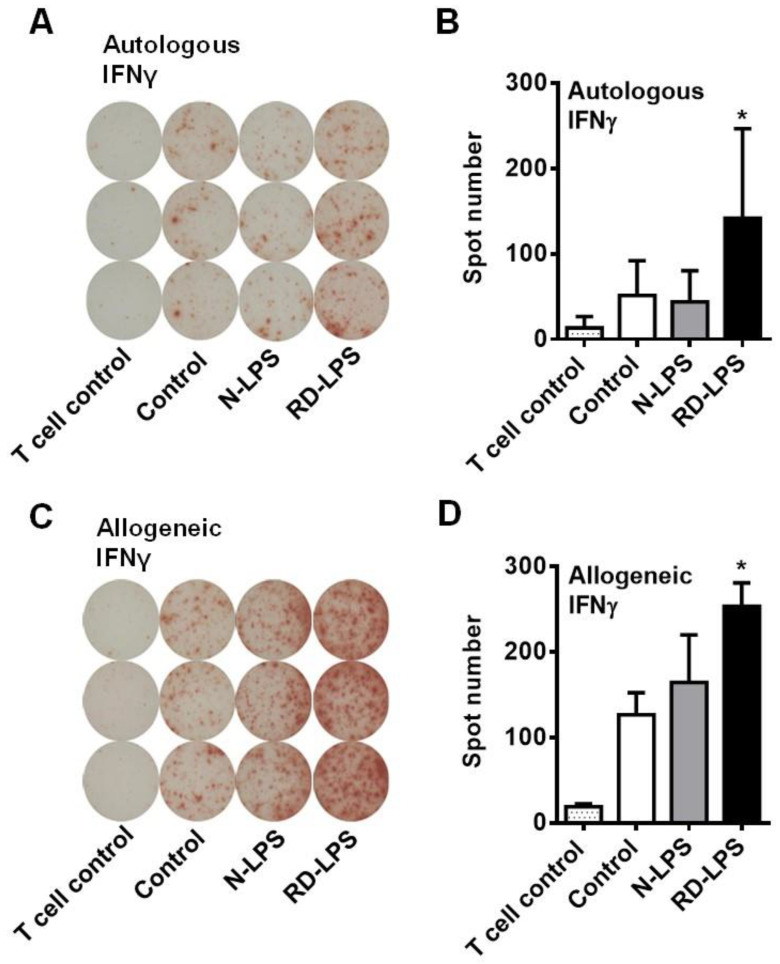
Radiation-detoxified LPS (RD-LPS) has a higher potential to induce Th_1_ polarization than native LPS (N-LPS). Human moDCs were treated with 250 ng/mL of the different endotoxins for 24 h, and then cells were co-cultured with autologous (**A**,**B**) or allogeneic (**C**,**D**) CD3^+^ pan-T-cells, respectively, for 4 days. After co-culturing, the formation of IFNγ-producing T-cells was determined by human IFNγ ELISPOT assay. Representative ELISPOT assays from four independent experiments in three parallel rows are shown in (**A**,**C**), where spots indicate individual IFNγ-producing T-cells. Data are shown as means ± SD from 4–8 independent experiments in (**B**,**D**). * *p* < 0.05 vs. control.

Previous data demonstrated that myeloid dendritic cells (DCs) in the airways play a pivotal role in sensitization to inhaled antigens and the development of Th_2_-dependent airway eosinophilia [45]. Therefore, in further experiments, we aimed to investigate the effects of N-LPS and RD-LPS on DC-mediated T-cell polarization using an in vitro experimental system (Figure 2A–D). To compare the T-cell-stimulatory capacity of N-LPS- and RD-LPS-treated moDCs, endotoxin-exposed DCs were co-cultured with autologous or allogeneic CD3^+^ pan-T-cells and after 4 days cytokine producing profile of T-cells was assessed by enzyme-linked immunospot (ELISPOT) assay. We found a significant difference between the two types of endotoxin only in the number of interferon (IFN) γ-producing cells. Stimulation of moDCs with N-LPS did not alter their capacity to activate autologous IFNγ-producing T-cells (Figure 2A,B), while it slightly, but not significantly, increased their ability to induce IFNγ secretion by allogeneic T-cells (Figure 2C,D). In contrast, treatment of moDCs with RD-LPS significantly enhanced the frequency of IFNγ-producing T-cells in both autologous and allogeneic co-cultures (Figure 2A–D). This T-cell-polarizing effect of RD-LPS could indicate an increased attenuating action on allergic reactions found in vivo.

### 2.3. N-LPS and RD-LPS Induce the Maturation of Human moDCs in a Similar Manner

**Figure 3:**
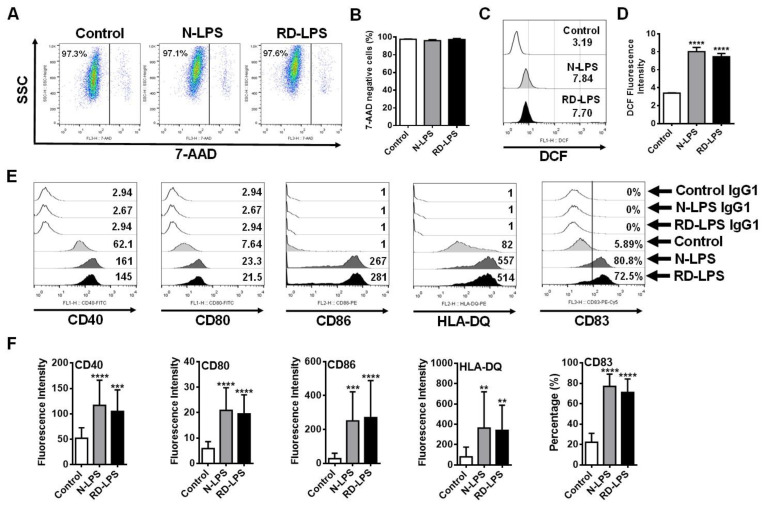
Human moDCs exposed to radiation-detoxified LPS (RD-LPS) exhibit similar viability parameters, intracellular ROS production and phenotypic changes as those treated with native LPS (N-LPS). (**A**–**F**) moDCs were treated with 250 ng/mL N-LPS or RD-LPS separately. (**A**,**B**) After 24 h, cell viability was measured by 7-aminoactinomycin D (7-AAD) staining using flow cytometry. Representative dot plots are shown in (**A**), where numbers indicate the percentage of 7-AAD negative cells. (**C**,**D**) To detect the intracellular ROS level in endotoxin-treated moDCs, 2′,7′-dihydrodichlorofluorescein diacetate (H_2_DCFDA)-loaded cells were used and exposed to the two types of endotoxin for 2 h. Flow cytometric analysis was applied to detect the changes in DCF fluorescence intensities, which correlate with the intracellular ROS accumulation. Representative histograms are shown in (**C**), and the numbers indicate the DCF fluorescence intensities. (**E**,**F**) The changes in the expression level of cell surface molecules were assessed at 24 h of endotoxin stimulation using flow cytometry. (**E**) Representative histograms are shown, where numbers indicate fluorescence intensities or the percentage of positive cells. (**B**,**D**,**F**) Bar graphs represent the means ± SD of 3–18 individual experiments. (**B**) ** *p* < 0.01, *** *p* < 0.001, **** *p* < 0.0001 vs. control.

Next, we wanted to explore the cause of the higher Th_1_-polarizing capacity of RD-LPS-exposed moDCs. It was a crucial point in the study that RD-LPS was found to be non-toxic to mice, unlike the parental LPS, which showed detectable toxicity. With the administration of RD-LPS to mice (8 mice per group) at a dose of 30 mg/kg, no animals died after 48 h (lethal dose (LD) of 30 mg/kg for RD-LPS = LD0). In contrast, N-LPS exposure resulted in the death of two out of eight mice at the same dose after 48 h (LD of 30 mg/kg for RD-LPS = LD25). Furthermore, the results of the limulus amoebocyte lysate (LAL) assay also demonstrated that limulus reactivity of the RD-LPS was abolished compared to N-LPS (limulus reactivity: RD-LPS/N-LPS = 1/1000). Therefore, first, we also wanted to test the effects of endotoxins on the viability of human moDCs to exclude impaired DC functions due to the toxic effect of N-LPS. Our results demonstrated that none of the endotoxin treatments affected the viability of moDCs at the applied dose (250 ng/mL) (Figure 3A,B). Endogenously generated reactive oxygen species (ROS) are essential mediators in antigen presentation by DCs [46]; thus, the intracellular ROS-inducing capacity of N-LPS and RD-LPS was also determined using a redox-sensitive fluorescence dye (H_2_DCF-DA). Treatments of moDCs with either N-LPS or RD-LPS resulted in a significant increase in the DCF fluorescence (Figure 3C,D). However, there were no significant differences in DCF fluorescence intensities between N-LPS- and RD-LPS-treated cells (Figure 3C,D), indicating the same intracellular ROS-inducing capacity of the two different types of LPS. Next, we examined how gamma-ray irradiation affects the ability of LPS to induce the phenotypic maturation of moDCs, which is required for T-cell interaction [47,48,49]. To assess the phenotypic changes in the cells, the expressions of various cell surface proteins including the maturation marker CD83, the major antigen-presenting molecule HLA-DQ, and the co-stimulatory molecules CD40, CD80 and CD86 were measured by means of flow cytometry (Figure 3E,F) using an appropriate gating strategy (Supplementary Figure S1). Our results demonstrate that both N-LPS and RD-LPS induced a significant increase in the expression of all cell surface proteins as compared to untreated control cells. However, no statistically significant differences were found between the moDC-activating potential of the two types of LPS (Figure 3E,F). These data indicate that N-LPS and RD-LPS induce phenotypic maturation in human moDCs to the same extent; therefore, the higher Th_1_-polarizing capacity of RD-LPS-exposed moDCs cannot be explained by a different ability of endotoxins to induce DC maturation.

### 2.4. RD-LPS-Exposed Human moDCs Are Characterized by a Reduced Ability to Produce Various Cytokines Compared to Those Treated with the Native Form

**Figure 4:**
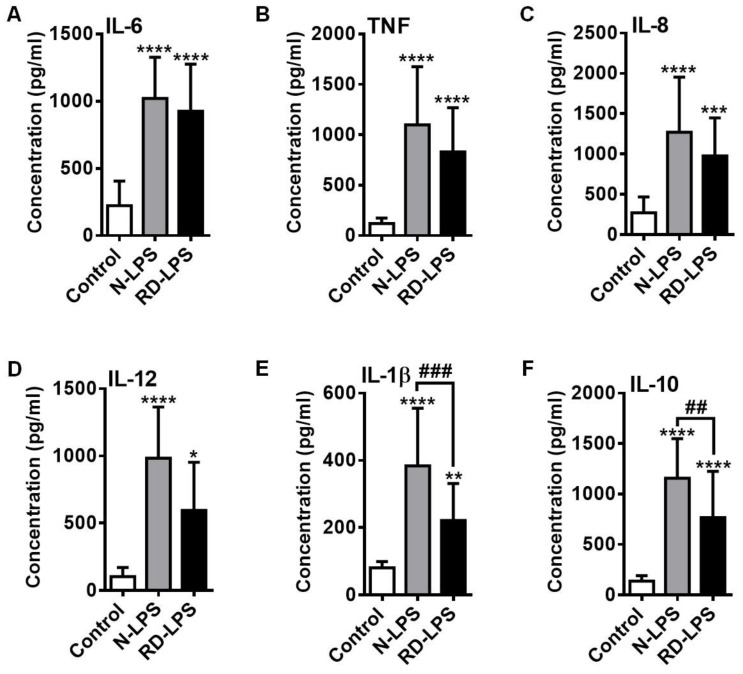
Cytokine and chemokine profile of native LPS (N-LPS)- and radiation-detoxified LPS (RD-LPS)-treated human moDCs. (**A**–**F**) Human moDCs were treated with the two different types of endotoxin for 24 h. After treatments, the concentrations of the secreted cytokines (**A**) IL-6, (**B**) TNFα, (**D**) IL-12, (**E**) IL-1β, and (**F**) IL-10 and the chemokine (**C**) IL-8 were determined from the supernatant of the cell cultures by means of ELISA. Data are presented as means ± SD of 8–17 independent experiments. * *p* < 0.05, ** *p* < 0.01, *** *p* < 0.001, **** *p* < 0.0001 vs. control, ## *p* < 0.01, ### *p* < 0.001 vs. N-LPS.

To determine the cytokine and chemokine profile of endotoxin-treated human moDCs, the concentrations of secreted pro-inflammatory cytokines IL-6, TNFα, IL-12 and IL-1β (Figure 4A,B,D,E), the anti-inflammatory cytokine IL-10 (Figure 4F) and the chemokine IL-8 (Figure 4C) in the supernatants of the cell cultures were measured via Enzyme-linked immunosorbent assay (ELISA). Similar to the induction of phenotypic changes, both forms of LPS were able to significantly increase the secretion of all of the measured cytokines and chemokines derived from moDCs (Figure 4A–F). Although lower cytokine and chemokine concentrations were detected in the supernatant of RD-LPS-treated moDCs than in those treated with the native form of LPS, significant differences between the effects of the irradiated and parental LPS could only be assessed in the levels of IL-1β (Figure 4E) and IL-10 (Figure 4F).

**Figure 5:**
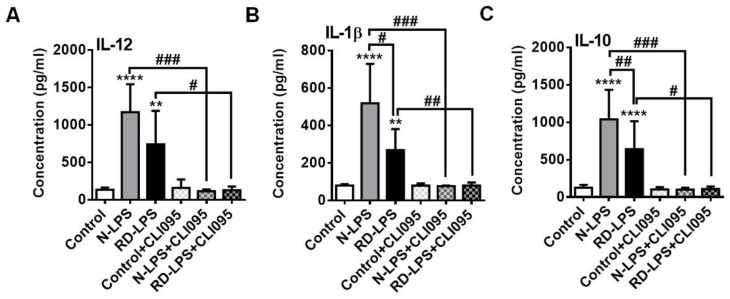
The effect of both native LPS (N-LPS) and radiation-detoxified LPS (RD-LPS) on moDCs is abolished in the presence of a specific TLR4 inhibitor. Human moDCs were pre-treated with 1 µM of specific TLR4 inhibitor (CLI-095) for 1 h and subsequently stimulated with 250 ng/mL of N-LPS or RD-LPS for 24 h. The levels of the secreted cytokines (**A**) IL-12, (**B**) IL-1β and (**C**) IL-10 were detected from the supernatants of the cell cultures by ELISA. Data are presented as means ± SD of 4 independent experiments. ** *p* < 0.01, **** *p* < 0.0001 vs. control, # *p* < 0.05, ## *p* < 0.01, ### *p* < 0.001 vs. N-LPS or RD-LPS.

Furthermore, we also demonstrated that protein secretion induced by both N-LPS and RD-LPS in moDCs was Toll-like receptor (TLR) 4-dependent (Figure 5A–C). When cells were pre-treated with CLI-095, also known as TAK-242, an intracellular TLR4 antagonist [50], the specific inhibition of TLR4 signaling completely abrogated endotoxin-induced production of IL-12 (Figure 5A), IL-1β (Figure 5B) and IL-10 (Figure 5C) in moDCs. Since the release of cytokines after the blockade of TLR4 remained at the basal levels in response to either N-LPS or RD-LPS, we may conclude that the radiation-detoxified form of the endotoxin was also able to react with TLR4 and retained its binding specificity to this receptor.

### 2.5. Both N-LPS and RD-LPS Induce Endotoxin Tolerance in Human moDCs

**Figure 6:**
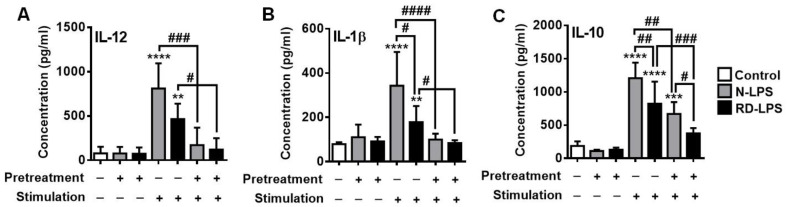
Cytokine profile of human endotoxin-tolerant human moDCs. Human moDCs were exposed to 5 ng/mL of native LPS (N-LPS) or radiation-detoxified LPS (RD-LPS) on the 4th day of DC differentiation and after 48 h (on day 6th), the cells were re-activated with 250 ng/mL of the different endotoxins for 24 h, generating endotoxin tolerance in the cells. After treatments, the levels of the secreted (**A**) IL-12, (**B**) IL-1β and (**C**) IL-10 cytokines were measured from the supernatant of the cell cultures by ELISA. Data are presented as means ± SD of 5–14 independent experiments. ** *p* < 0.01, *** *p* < 0.001, **** *p* < 0.0001 vs. control, # *p* < 0.05, ## *p* < 0.01, ### *p* < 0.001, #### *p* < 0.0001 vs. N-LPS or RD-LPS.

Endotoxin tolerance or LPS tolerance is a protective mechanism in which cells or organisms exposed to low concentration of LPS enter a transient state and display reduced sensitivity to respond to a second LPS challenge [51]. In the next set of experiments, we investigated the ability of N-LPS and RD-LPS to induce endotoxin tolerance in human moDCs (Figure 6A–C). On day 4 of differentiation, human moDCs were pre-treated with a low dose of LPS (5 ng/mL) and re-exposed to a high dose of LPS (250 ng/mL) on day 6. After 24 h of treatment, the concentrations of IL-12 (Figure 6A), IL-1β (Figure 6B) and IL-10 (Figure 6C) were determined from the supernatants of cell cultures by ELISA. Under our experimental conditions, significant reductions in cytokine production were detected upon LPS restimulation of moDCs compared to a single high-dose LPS treatment, indicating the development of endotoxin tolerance in moDCs. Restimulation of moDCs with N-LPS and even RD-LPS resulted in a significantly lower concentration of IL-12, IL-1β and IL-10 in the supernatants of the cells compared to a single high-dose exposure (Figure 6A–C). However, there were no significant differences in the amount of released cytokines between N-LPS-re-activated moDCs and RD-LPS-re-activated ones, except in the case of IL-10 (Figure 6C). Both single-dose and repeated N-LPS treatments induced significantly higher levels of IL-10 production than exposure to RD-LPS (Figure 6C). These findings show that both the RD-LPS-activated and the RD-LPS-tolerant moDCs have a lower capacity to produce the IL-10 cytokine than cells exposed to the native form of the endotoxin.

### 2.6. The Myristic Acid Content of Radiation-Detoxified Endotoxin May Influence Its Immunomodulatory Effects

**Figure 7:**
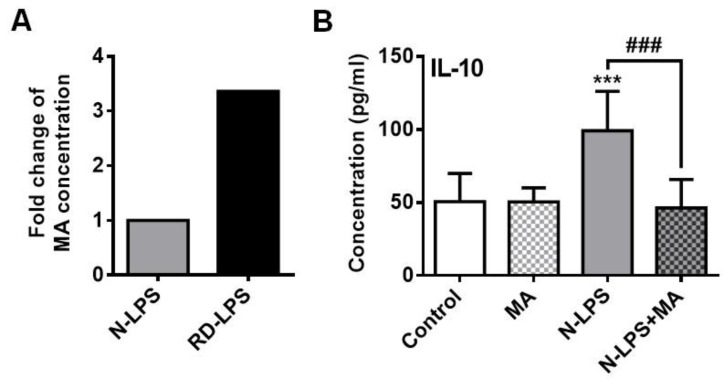
Myristic acid affects the endotoxin-induced IL-10 production of human moDCs. (**A**) Myristic acid content of native LPS (N-LPS) and radiation-detoxified LPS (LPS) preparations was determined by means of high performance liquid chromatography- mass spectrometry (HPLC-MS). Bar graph indicates fold changes. (**B**) Human moDCs were exposed to 250 ng/mL native LPS (N-LPS) in the absence or presence of myristic acid (MA) for 24 h and the level of secreted IL-10 was measured from the supernatant of the cell cultures by ELISA. Data are presented as means ± SD of 8 independent experiments. *** *p* < 0.001 vs. control, ### *p* < 0.001 vs. N-LPS.

Differences in the immunological profile of the two endotoxin preparations may be due to the degradation of endotoxin molecules by radiation, resulting in a series of various free fatty acids bearing immunomodulatory properties. In our preparations, the concentration of β-hydroxymyristic acid, determined by high performance liquid chromatography-mass spectrometry (HPLC-MS), was threefold higher in RD-LPS (1.82 µg/mL in 1 mg/mL LPS) than that in N-LPS (0.53 µg/mL in 1 mg/mL LPS) (Figure 7A). Based on these findings, we supposed that the higher concentration of the myristic acid derivative found in RD-LPS may affect the IL-10-inducing potential of moDCs exposed to RD-LPS, leading to lower IL-10 production. To test this hypothesis, we investigated the IL-10 secretion of moDCs exposed to the native form of endotoxin with lower myristic acid content in the presence of experimentally added myristic acid (Figure 7B). The myristic acid treatment alone did not alter the basal level of IL-10 in moDCs, but completely inhibited N-LPS-induced IL-10 secretion by the cells (Figure 7B). Thus, the higher level of myristic acid in the RD-LPS preparation might be responsible for the impaired IL-10 production of RD-LPS exposed moDCs, and at lower IL-10 levels, the polarization towards Th_1_ may be more pronounced.

## 3. Discussion

Lipopolysaccharide molecules are the major outer surface membrane components present in most Gram-negative bacteria and act as potent activators of innate immune responses. The basic molecular structure of LPS consists of two distinct regions: a hydrophilic polysaccharide portion, which includes an O-specific side chain and an inner and outer core region, and an amphipathic, endotoxically active lipid A component [52,53]. Lipid A represents the conserved molecular pattern of LPS and is the main inducer of immunological responses to LPS. Prior studies reported that ionizing radiation modifies both physical and biological properties of LPS, leading to reduced toxicity [42,54]. Furthermore, studies on animals and human have shown that exposure to ^60^Co-radiation preserves the capacity of LPS to function as an immunoadjuvant and immunomodulator, to protect against endotoxin shock and radiation-induced disease and to stimulate natural resistance (reviewed in [43]). In this study, we investigated whether radio-detoxified LPS in the form of environmental aerosol spray can be used as a prophylactic agent in pollen-induced allergic reactions.

Previous studies indicated that the detoxification effect of ionizing radiation is probably due to the destruction of polysaccharide moieties and possibly the alteration of lipid A component of the LPS molecules. RD-LPS preparations showed a significant reduction in glucosamine, phosphate, 2-keto-3-deoxyoctonic acid, and fatty acid components, indicating an alteration in the lipid A structure compared to parental LPS preparations [54,55]. The structure–activity relationship of the lipid A portion of LPS has been extensively studied and the total number of lipid chains has been identified as the most important factor governing the immunological activity of LPS [56]. Lipid A with six lipid chains has optimal inflammatory activity, while lipid A molecules with five lipid chains are ~ 100-fold less active, and those with four lipid chains completely lack agonistic activity [56]. Our results, showing that limulus reactivity of the LPS was also destroyed by ionizing radiation, are in line with previous observations [54]. The authors of that prior study hypothesized that the decreased LAL reactivity of LPS upon exposure to ionizing radiation was associated with molecular changes, notably with the removal of polysaccharide components and/or partial destruction of the lipid A component [54]. These data highlight that while the endotoxically active part of the molecule is lipid A, the nature and number of attached saccharide residues and substituents do have a considerable impact on modulating this activity (as reviewed in [57]).

Several previous studies have investigated how LPS treatment of experimental animals can influence the outcome of allergen-induced immune responses. In these studies, there were many variations in the source and concentration of allergens used, the amount of contaminating or intentionally added LPS, and in the protocols for allergen sensitization and challenge (reviewed in [26]). Even the sampling time of the airway inflammation and the pulmonary function test after the allergen challenge could differ from one study to another. These could be the reasons for the confusing, sometimes contradictory reports on the effects of LPS on allergen-induced responses from different laboratories [26].

Our experiments were designed to model the hygiene hypothesis that early exposure to high levels of innate immune-stimulating compounds such as endotoxins may protect against the development of atopic diseases [58,59]. Endotoxins are continuously shed from the outer membrane of bacteria and released into the environment, and thus endotoxins can be found anywhere that bacteria are present, including dust, air, water, soil, drinks or food [13]. Therefore, in our experiments, we applied LPS in an aerosol form prior to the allergen challenge, which allowed the endotoxin to be distributed everywhere in the environment of the laboratory animals, including bedding, food and drink. This technique helped us to better promote the renaturation of the environment and mimic conditions on farms, where LPS levels are significantly higher than in inner-city homes [14]. Thus, endotoxin uptake by mice during treatments could be achieved not only by inhalation but also orally. Both entry routes of endotoxin have been described to contribute to the prevention of atopy [15,25,60]. However, it is important to highlight that the time-dependent effects of endotoxin or farm exposure are strongest in two critical windows: prenatal and early childhood [61]. In most studies, this early childhood window refers to the first 3 weeks in mice and the first year of life in humans [61,62]. However, some studies have shown that the protective effects of LPS exposure in mice can persist into adulthood [18,63]. In our experiments, we observed the preventative effects of endotoxin pre-exposure on pollen-induced allergy in six-week-old mice. Moreover, contrary to previous studies, we did not detect a protective effect of the native form of this endotoxin on allergic inflammation. Only the detoxified form proved to be an effective prophylactic agent. Indeed, in our experimental system, only prolonged pre-treatment with RD-LPS was able to significantly reduce the ragweed pollen-induced airway eosinophilia compared to the native endotoxin. This result suggests that the radiation-detoxified form of LPS may be a more effective and safer approach to prevent atopy.

To explore the factors that may have contributed to the more effective ability of RD-LPS to attenuate Th_2_-type airway inflammation, we compared the immunomodulatory properties of RD-LPS and parental N-LPS on human moDCs. As professional antigen-presenting cells, DCs play an essential role in the activation of T-cells and the polarization of T-cell-mediated adaptive immune responses [64]. Data from ELISPOT measurements indicated that treatments of moDCs with RD-LPS significantly increased the frequency of IFNγ-producing Th_1_ cells in co-cultures compared to N-LPS. This Th_1_-cell-polarizing effect of RD-LPS could suggest an enhanced ability to attenuate allergic reactions in vivo [65]. Previous observations revealed significant differences in the effects of N-LPS and RD-LPS, which can be attributed to the changes in the chemical structure induced by ionizing radiation [43,66]. Generally, the composition of lipid A in LPS shows minor variations and is consequently a shared, conserved component of all bacterial endotoxins. In contrast, the R-core and O-antigen display significant polymorphism in carbohydrate composition [67]. Bacteria with an O-antigen in their LPS structure are designated as smooth (S) strains, while those lacking this component are classified as rough (R) strains. Bacteria with only one O-chain repeating unit are known as semi-rough (SR) strains [68]. The lipid A component serves as the active, pro-inflammatory part of LPS molecules, directly binding to TLR4 and MD2 [56]. Consequently, changes or variations in lipid A structure are known to alter the pro-inflammatory features of LPS [69]. Apart from the lipid A structure, the length of the polysaccharide chain could also partially explain the variability in the inflammatory activity of LPS. It was previously published that rough LPS, characterized by a short polysaccharide chain, elicits weaker pro-inflammatory (IL-6, TNF) and anti-inflammatory cytokine (IL-10) responses compared to smooth LPS with a longer polysaccharide chain [70]. Our results demonstrate that RD-LPS-treated moDCs exhibited a decreased tendency in the concentrations of all measured secreted cytokines compared to N-LPS. The reduced pro-inflammatory activity of RD-LPS is likely due to the alterations in the incomplete lipid A and R core segments induced by ionizing radiation, as well as the removal of the O-polysaccharide side chain unit [71,72]. These changes may result in a structure similar to rough LPS, which is also characterized by reduced inflammatory potential [70].

Among the examined moDC-derived cytokines, RD-LPS resulted in significantly lower levels of proteins only in the case of the pro-inflammatory cytokine IL-1β and the anti-inflammatory cytokine IL-10 compared to N-LPS. It was previously reported that IL-1β significantly enhances CD4 T-cell survival, antigen-driven expansion, differentiation, and in vivo cytokine production [73]. Together with IL-23, it induces the expansion and commitment of naïve and memory CD4+ T-cells into Th_17_ in response to antigens, and enhances their function [74,75]. In addition, it has also been observed that IL-1β has an impact on Th_2_ differentiation [76]. Culturing CD4 T-cells under Th_2_-polarizing conditions in the presence of IL-1β resulted in a significant increase in IL-13 production compared to cells without IL-1 signaling, which were used as a control. Additionally, as anticipated based on the increased IL-13 production, the number of infiltrating eosinophils was elevated in the BALF of IL-1β-treated and allergen-challenged mice, along with an increase in mucus production and goblet cell metaplasia [76]. These results indicate that the presence of IL-1β during in vivo Th_2_ priming leads to increased allergic effector responses. From these results, we can conclude that RD-LPS, with lower IL-1β inducing capacity compared to N-LPS, could maintain lower levels of IL-13, limiting the infiltration of eosinophils after allergen challenge.

IL-10 has been reported as a global suppressor of immune responses and a regulator of the Th cell responses [77]. Nevertheless, the immunoregulatory function of IL-10 seems to be more complex. IL-10 was originally described as a product of Th_2_ cells capable of suppressing the cytokine production of Th_1_ cells [78]. Later, it was revealed that monocytes and macrophages [78], as well as DCs [79], can secrete a significant amount of IL-10 in response to an LPS stimulus. Our results suggest that the IL-10-inducing effect was more pronounced for N-LPS than for RD-LPS, which may explain the lower Th_1_-polarizing capacity of N-LPS, possibly impaired in the presence of higher IL-10 levels. In addition, DCs producing IL-10 have been shown to promote the development of Th_2_ cells in vitro [80,81]. Moreover, the IFNγ-limiting and Th_2_-promoting roles of IL-10 have been previously described in various Th_2_-type inflammations using different in vivo mouse models [82,83]. It is important to note that in our experimental system, both RD-LPS and N-LPS were able to induce endotoxin tolerance, as they both inhibited the production of IL-12 and IL-1β pro-inflammatory cytokines after repeated LPS treatment. The fact that RD-LPS preserved its ability to induce endotoxin tolerance is also considered a beneficial property of the detoxified LPS, in addition to its non-toxic nature. As has been previously described, the development of endotoxin tolerance in the body can be a crucial factor in attenuating allergic airway inflammations [84,85]. In addition to the reprogramming of endotoxin-tolerant cells to produce fewer pro-inflammatory molecules upon secondary stimulation, these cells also generate elevated levels of anti-inflammatory cytokines, such as IL-10 and transforming growth factor beta (TGFβ), compared to the levels produced by naive cells [86,87]. However, in our system, neither the native nor the radiation-detoxified form of LPS resulted in increased IL-10 levels in endotoxin-tolerant moDCs compared to cells treated with a single LPS dose. The observed difference is likely due to the heterogeneity of LPS molecules, as the bacterial species from which the LPS originates determines the quality of the immune responses [68].

Furthermore, our results suggest that the IL-10 production of RD-LPS-exposed moDCs might have been affected by free fatty acids generated through ionizing radiation of the endotoxin, as the concentration of β-hydroxymyristic acid was threefold higher in the RD-LPS preparations than in N-LPS. It is widely accepted that free fatty acids have immunomodulatory properties and influence the outcomes of immune responses in the body [88]. Myristic acid has previously been described to have antifungal [89,90] and anti-inflammatory effects [91]. Additionally, myristic acid has been identified as a metabolic checkpoint that regulates autophagy and type I IFN responses [92]. In the present study, we confirmed that the IL-10 production of LPS-treated moDCs can be inhibited in the presence of myristic acid. Thus, we can conclude that the higher myristic acid content in the RD-LPS preparations may have contributed to the lower IL-10 production of RD-LPS-exposed moDCs compared to N-LPS-exposed ones. Due to the lower IL-10 levels, RD-LPS-exposed moDCs had a greater capacity for inducing IFNγ-producing Th_1_ cells in comparison to DCs treated with the native form of LPS.

**Figure 8:**
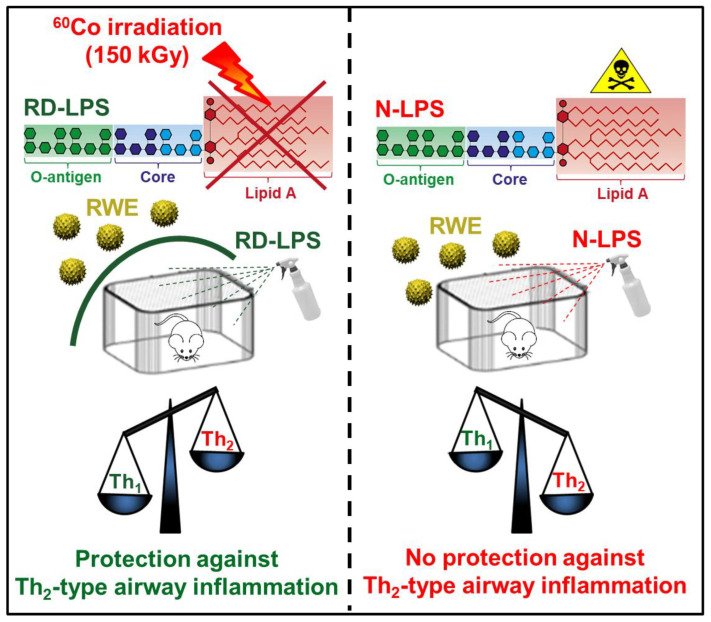
Renaturation of the environment by the radiation-detoxified form of lipopolysaccharide (RD-LPS) can offer a safe and effective solution to prevent allergic inflammation. The irradiation of endotoxin results in a safer and non-toxic form of LPS by destroying the structural elements responsible for its toxic effects while preserving its immunomodulatory properties. Using RD-LPS in the form of an aerosol spray for environmental renaturation may help to establish an allergy-protective environment, contributing to a reduction in allergic diseases. In contrast to the native form of LPS (N-LPS), RD-LPS is an effective inducer of the Th_1_ responses, and this shift in T-cell polarization can provide protection against the development of Th_2_-dominant allergic inflammations. Co: cobalt, N-LPS: native lipopolysaccharide, RD-LPS: radiation-detoxified lipopolysaccharide, RWE: ragweed pollen extract, Th: T helper.

In conclusion, our results demonstrate that the long-term application of non-toxic RD-LPS before an airway allergen challenge has significant protective effects on allergic airway inflammations in mice compared to the parental LPS. This ability of RD-LPS is likely due to its greater Th_1_-polarizing effect compared to parental LPS, which aids in the prevention of or reduction in Th_2_-type allergic inflammation by establishing Th_1_ dominance. Therefore, in contrast to the native and toxic forms of LPS, the better anti-allergic capabilities of non-toxic RD-LPS in the form of an aerosol represent a potential new approach to prevent or attenuate allergic inflammations (Figure 8). Regular application of RD-LPS in the form of an aerosol can contribute to the restoration of the urban environment, particularly in the sleeping rooms of infants. However, it could be effective only in the first year of life when the window of susceptibility to external microbial stimuli is suggested to exist [35].

In summary, our findings can encourage new pharmaceutical and clinical studies to test the aerosol preparations of RD-LPS for the prevention of various allergic diseases starting in childhood.

## 4. Materials and Methods

### 4.1. Bacterial Endotoxin Preparations

For in vivo as well as in vitro experiments, N-LPS was extracted from Escherichia coli serotype O101RG/W using the hot aqueous-phenol method [93]. This method provides high-quality LPS extracts away from other cellular components, including nucleic acids and proteins as previously published [94]. Following fractionation of the LPS isolates by means of SDS-PAGE electrophoresis, endotoxins were visualized by standard silver staining, and Coomassie Blue staining was used to exclude protein contamination, while ethidium bromide dye was used to exclude nucleic acid contamination as described previously [95]. The extracted N-LPS was detoxified by ^60^Co irradiation with under 150 kGy (Lot. number: 92040605/K/01; National Research Institute for Radiobiology and Radiohygiene, Budapest, Hungary). Toxicity of N-LPS and RD-LPS was tested on BALB/c mice (8 mice per group) after 48 h of exposure: LD of 30 mg/kg for N-LPS= LD25 and for RD-LPS= LD 0. The ratio of endotoxin activities was tested via LAL assay (ThermoFisher Scientific, Waltham, MA, USA): RD-LPS/N-LPS = 1/1000.

### 4.2. In Vivo Mice Experiments

Cages (feed and bedding) of six-week-old female BALB/c mice (4–10 per group) were treated daily for 70 days with 5 µg of N-LPS or RD-LPS dissolved in 5 mL of endotoxin-free water (Cat. no.: TMS011; Sigma-Aldrich, St. Louis, MO, USA) in the form of an aerosol spray. As a control, only endotoxin-free water was used. To maintain the endotoxin-free status of the ultra-pure water provided by the manufacturer (endotoxin < 0.005 EU/mL), the dissolving of LPS was always freshly performed before treatments under sterile conditions using autoclaved (at 121 °C and 15 psi for 60 min) sterile laboratory equipment. The optimal dose of the endotoxin was calculated according to a previous publication [96]. During a 70-day LPS treatment period, mice were sensitized intraperitoneally with two injections of 150 µg endotoxin-free ragweed pollen extract (RWE; Greer Laboratories, Lenoir, NC, USA) adsorbed with 150 µg of aluminum hydroxide in 100 µL of phosphate-buffered saline (PBS; PAA Laboratories GmbH, Pasching, Austria) on days 60 and 64. On day 71, when the LPS treatments were finished, the allergen challenges were performed intranasally using 100 µg of RWE. Then, 72 h after allergen challenge, mice were sacrificed and inflammatory cells from the bronchoalveolar lavage fluid (BALF) were analyzed. Tracheas of euthanized mice were cannulated and the lavages were performed using 2 aliquots of 0.7 mL of PBS (PAA Laboratories GmbH). The BALF samples were centrifuged at 400× *g* for 10 min at 4 °C to sediment the cells. After the supernatants were removed, cells were suspended in PBS. After cyto-centrifugation, the preparations were stained with Wright–Giemsa and the cell counts were calculated for each BALF. On the stained slides, the number of eosinophils, neutrophils, lymphocytes, and macrophages were determined by counting at least 400 cells. The above-described murine model of allergic airway inflammation was established according to previous publications [97,98,99,100]. In this model, the use of an endotoxin-free form of RWE was crucial, as ensured by the manufacturer, because we aimed to establish a mouse model of type 2 allergic inflammation, in which eosinophils are the dominant cell types, similar to human eosinophilic asthma [101]. Previously, it was observed that endotoxin contamination of the allergen extract can alter the cellular inflammation during the late airway response following allergen challenge and may facilitate the recruitment of neutrophils rather than eosinophils [102]. Thus, the endotoxin content of the RWE stock solutions was consistently monitored using LAL assay (ThermoFisher Scientific), which was found to be negligible, as we previously reported [103].

The care and handling of animals followed the Helsinki Declaration, European Union regulations and adhered to the guidelines of the Committee for Research and Ethical Issues of IASP. All animal study protocols were approved by the Animal Care and Protection Committee at the University of Debrecen (#7/2011/DE MAB). Animals were maintained in the pathogen-free animal facility of the University of Debrecen. All day food and water were available ad libitum.

### 4.3. Isolation of Human Cells

Human cells were isolated from heparinized leukocyte-enriched buffy coats drawn from healthy donors at the Regional Blood Center of Hungarian National Blood Transfusion Service (Debrecen, Hungary), with the written approval of the Director of the National Blood Transfusion Service and the Regional and Institutional Ethics Committee of the University of Debrecen, Faculty of Medicine (Debrecen, Hungary). The collection of human samples complied with the guidelines of the Helsinki Declaration. First, peripheral blood mononuclear cells (PBMCs) were isolated by means of Ficoll–Paque (GE Healthcare, Uppsala, Sweden) density gradient centrifugation of buffy coats of healthy volunteers. Then, human monocytes and human CD3+ pan-T-cells were separated from PBMCs by positive selection using immunomagnetic anti-CD14 and anti-CD3 microbeads (both from Miltenyi Biotec, Bergisch-Gladbach, Germany), according to the manufacturer’s instructions. The purity of the monocyte and even CD3+ T-cell fraction was over 97%, as determined by flow cytometry.

### 4.4. Generation of moDCs from Human CD14+ Monocytes

Freshly isolated monocytes were cultured in 24-well tissue culture plates at a density of 1 × 10^6^ cells/mL in RPMI 1640 medium (Sigma-Aldrich) supplemented with 2 mM L-glutamine (Sigma-Aldrich), 100 U/mL penicillin, 100 ng/mL streptomycin, and 10% heat-inactivated FBS (Life Technologies Corporation, Carlsbad, CA, USA). For stimulation, 80 ng/mL granulocyte-macrophage colony-stimulating factor (Gentaur Molecular Products, Brussels, Belgium) and 100 ng/mL IL-4 (Peprotech EC, London, UK) were added to the cells immediately and 48 h later. Cells were cultured at 37 °C in 5% CO_2_ humidified atmosphere and were used for experiments on day 5, when more than 90% of cells showed an immature DC phenotype (DC-SIGN/CD209+, CD14low).

### 4.5. In Vitro Treatments Conditions

Human 5-day-old moDCs were seeded at 1 × 10^6^ cells/well in 24-well plates in RPMI 1640 medium (Sigma-Aldrich) supplemented with 2 mM L-glutamine (Sigma-Aldrich), 100 U/mL penicillin, 100 ng/mL streptomycin, 10% heat-inactivated FBS (Life Technologies Corporation). For stimulation, moDCs were treated with 250 ng/mL N-LPS or RD-LPS for 24 h. In parallel experiments, 5-day-old moDCs were pre-treated with 1 µM of a specific TLR4 inhibitor, CLI-095 (InvivoGen, San Diego, CA, USA). After 1 h of incubation with the inhibitor, cells were subsequently stimulated with 250 ng/mL N-LPS or RD-LPS for 24 h. To induce endotoxin tolerance in moDCs, cells were exposed to 5 ng/mL N-LPS or RD-LPS on the 4th day of DC differentiation, and after 48 h, the culture media of the cells were replaced and cells were re-activated with 250 ng/mL N-LPS or RD-LPS for 24 h. The IL-10 production of endotoxin-treated moDC was also investigated in the presence of myristic acid (300 µM; Sigma-Aldrich).

### 4.6. Assessment of Intracellular ROS Level in Human moDCs

Human 5-day-old moDCs were loaded with 50 μM of 2′,7′-dihydrodichlorofluorescein diacetate (H2DCFDA; Invitrogen, Waltham, MA, USA) for 25 min at 37 °C in the dark. After incubation, cells were washed twice with PBS (PAA Laboratories GmbH) to remove the excess fluorescent dye and then cells were exposed to endotoxins for 2 h. Changes in DCF fluorescence intensities were assessed with FACS Calibur Flow Cytometer (Becton Dickinson, Franklin Lakes, NJ, USA) on the FL1 (530 ± 15 nm) channel and data analysis was performed using FlowJo v. 5.7.2 Software (Treestar, Ashland, OR, USA).

### 4.7. Phenotypic Analysis of Human moDCs

The phenotypic analysis of the endotoxin-treated moDCs was performed by labelling the cells with the following monoclonal antibodies conjugated with the respective fluorescent dyes: anti-CD40-FITC, anti-CD83-PE-Cy5 (both from BD Pharmingen, San Diego, CA, USA), anti-CD80-FITC, anti-HLA-DQ-PE (both from BioLegend, Uithoorn, The Netherlands), anti-CD86-PE (R&D System, Minneapolis, MN, USA) and isotype-matched control antibodies, all from BD Pharmingen. Fluorescence intensities were detected via multicolor flow cytometry using a FACS Calibur Flow Cytometer (Becton Dickinson), and the data were analyzed by FlowJo v. 5.7.2 Software (Treestar).

### 4.8. Detection of Cytokine and Chemokine Release of Human moDCs

Enzyme-linked immunosorbent assays (ELISAs) were performed to quantify the secretion of cytokines and chemokines by moDCs. ELISA kits for IL-1β, IL-6, IL-8, IL-10, IL-12 and TNFα (all from BD Bioscience, San Diego, CA, USA) were used according to the manufacturer’s instructions. Absorbance measurements were obtained using a Synergy HT micro plate reader (Bio-Tek Instruments, Winooski, VT, USA) at 450 nm.

### 4.9. Enzyme-Linked Immunospot (ELISPOT) Assay

Human moDCs were exposed to 250 ng/mL N-LPS or RD-LPS for 24 h. After incubation, moDCs were washed twice with cell culture medium and were co-cultured with autologous or allogeneic human CD3+ pan-T-cells at a ratio of 1:10 (moDC/T-cell) in 48-well tissue culture plates for 4 days. After co-culturing, the number of IFNγ-producing T-cells was determined by using a commercial ELISPOT assay specific for human IFNγ (eBioscience, San Diego, CA, USA) according to the manufacturer’s instructions. Briefly, after a 4 day-long co-cultivation, T-cells were washed twice with PBS (PAA Laboratories GmbH) and plated in a serum-free test medium (Cellular Technology Ltd., Bonn, Germany) at 1 × 10^5^ cells/100 μL/well in polyvinylidene difluoride-coated 96-well MultiScreen Filter Plates (Millipore, Schwalbach, Germany) for 24 h. Plates were previously prepared by adding a capture antibody and by blocking with RPMI 1640 medium supplemented with 10% heat-inactivated FBS. After cultivation, the cells were removed from the plates and a detection antibody was added for 2 h. The plates were incubated for 45 min in the presence of avidin–horseradish peroxidase conjugate, and finally, BD ELISPOT AEC (3-amino-9-ethylcarbazole) substrate (BD Biosciences, Franklin Lakes, NJ, USA) was added and color change was allowed to develop for 5–30 min at room temperature, followed by rinsing with distilled water. The plates were dried completely, and spots were read on an ImmunoScan analyzer using ImmunoSpot 4.0 software (Cellular Technology Ltd.).

### 4.10. Ultra-High-Pressure Liquid Chromatography (UHPLC) Coupled to Electrospray Ionization Quadrupole Time-of-Flight Mass Spectrometry (ESI-QTOF-MS)

Separation and mass spectrometric detection of β-hydroxymyristic acid content in the LPS extracts were performed with an Infinity 1290 UHPLC (Agilent Technologies, Waldbronn, Germany) coupled to a 6530 Accurate Mass Q-TOF LC/MS system (Agilent Technologies, Singapore). First, free fatty acids were extracted from N-LPS and RD-LPS samples by adding 300 µL of water and 300 µL of hexane (VWR, Leuven, Belgium) to 1.5 mL Eppendorf tubes containing the 2 mg samples, respectively. The mixture was vortexed for 10 s and then centrifuged at 5600× *g* for 5 min. The hexane phase was collected into a separate Eppendorf tube and dried under N2. The extraction procedure was repeated 5 times. The collected sediment was dissolved in 50 µL of the mixture of acetonitrile/5 mM ammonium acetate (eluent B; 98:2 *v*/*v*%, pH 7.0, Sigma-Aldrich). Eluent A was 5 mM ammonium acetate (pH 5.0). Chromatographic separation was achieved using a fully porous Kinetex C18 column from Phenomenex (100 mm × 4.6 mm, 2.6 µm particle size). The injection volume of the sample solutions was 3 μL. Separation was performed at 40 °C at a constant flow rate of 0.5 mL/min. Elution was performed with a linear gradient from 70% to 100% mobile phase B over a set time of 7 min and the column was re-equilibrated for 5 min with 70% eluent B. Negative-ion mass spectra of the column eluates were recorded in the range of m/z 150–300 at a measuring frequency of 10,000 transients/s and a detection frequency of 4 GHz. The Agilent Jet Stream (AJS) ion source was operated using the following conditions: the pressure of nebulizing gas (N2) was 30 psi; the temperature and flow rate of drying gas (N2) was 325 °C and 8 L/min, respectively; and the temperature and flow rate of sheath gas was 300 °C and 11 L/min, respectively. The capillary voltage was set to 4 kV, the nozzle voltage was set to 1.8 kV, the fragmentor potential was set to 100 V and the skimmer potential to 65 V. A stock solution of the standard was prepared by dissolving the 1.0 mg β-hydroxymyristic acid standard (Sigma-Aldrich) in eluent B in a 10.0 mL volumetric flask. The working solutions were prepared by the dilution of the stock solution with eluent B. The concentrations of working solutions were 8.00, 4.00, 2.00, 1.00, 0.5, 0.25 and 0.13 µg/mL.

### 4.11. Statistical Analysis

Statistical significances were determined by one-way ANOVA with Bonferroni post hoc test for least-significant differences using the GraphPad Prism v.6. software (GraphPad Software Inc., La Jolla, CA, USA). Differences were considered to be statistically significant at *p* < 0.05.

## Data Availability

The data presented in this study are available in this article’s figures and in the Supplementary Materials. The data that support the findings of this study are available from the corresponding author upon reasonable request.

## Supplementary Materials

The following supporting information can be downloaded at: https://www.mdpi.com/article/10.3390/ijms25031581/s1.
