# Supplementary material for: Radiation-Detoxified Form of Endotoxin Effectively Activates Th1 Responses and Attenuates Ragweed-Induced Th2-Type Airway Inflammation in Mice
Source: Int J Mol Sci. 2024 Jan 27;25(3):1581. doi: 10.3390/ijms25031581 (PMC10855154; doi:10.3390/ijms25031581)
Supplement: Supplementary file 1 [file ijms-25-01581-s001.zip › ijms-2825487-supplementary.pdf]

International Journal of *Molecular Sciences*

MDPI

Supplementary Materials *To Lóránd Bertók's memory*

Radiation-detoxified form of endotoxin effectively activates Th1 responses and attenuates ragweed-induced Th2-type airway inflammation in mice

Attila Bácsi[1, †], Beatrix Ágics[1,2, †], Kitti Pázmándi[1], Béla Kocsis[3], Viktor Sándor[4], Lóránd Bertók[5, ‡], Geza Bruckner[6] and Sándor Sipka[7*]

[1] Department of Immunology, Faculty of Medicine, University of Debrecen, H-4032 Debrecen, Hungary; etele@med.unideb.hu (A.B.); agics.beatrix@med.unideb.hu (B.Á.); pazmandi.kitti@med.unideb.hu (K.P.)
[2] Doctoral School of Molecular Cell and Immune Biology, University of Debrecen, H-4032 Debrecen, Hungary
[3] Department of Medical Microbiology and Immunology, Faculty of Medicine, University of Pécs, H-7624 Pécs, Hungary; kocsis.bela@pte.hu
[4] Institute of Bioanalysis, Medical School and Szentágothai Research Center, University of Pécs, H-7624 Pécs, Hungary; sandor.viktor@pte.hu
[5] National Research Directorate for Radiobiology and Radiohygiene, National Public Health Center, H-1221 Budapest, Hungary; radbiol@osski.hu
[6] Department of Athletic Training and Clinical Nutrition, University of Kentucky, Lexington, KY 40536, USA; gbruckn@uky.edu
[7] Division of Clinical Immunology, Faculty of Medicine, University of Debrecen, H-4032 Debrecen, Hungary
* Correspondence: sipka.sandor45@gmail.com; Tel.: +36-52-255-212
† These authors contributed equally to this work.
‡ Deceased.

Supplementary Figure S1.

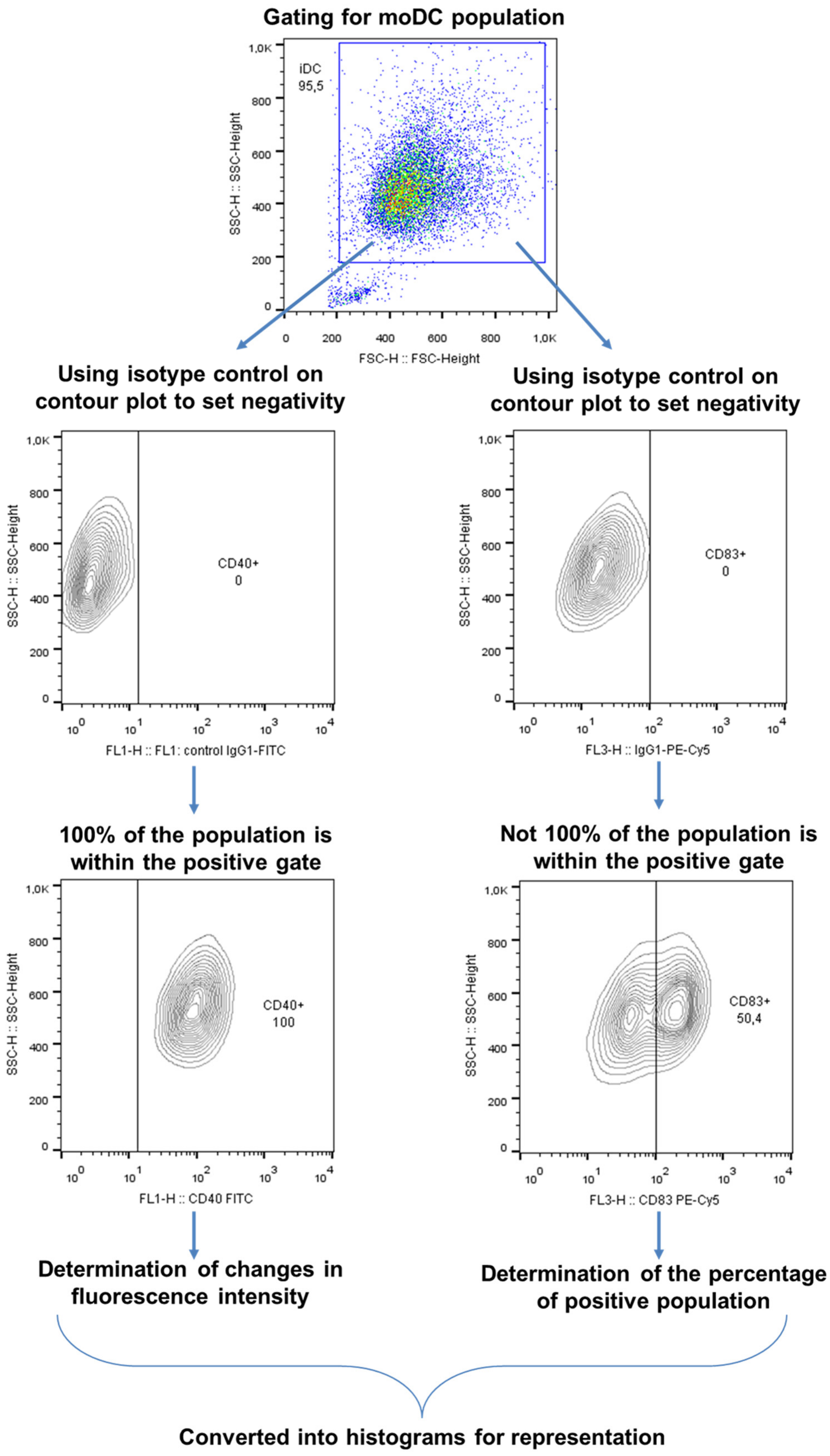

Supplementary Figure S1. Gating strategy of human moDCs for flow cytometric analysis. Representative dot plots and contour plots show the gating strategy to analyze the phenotypic changes of moDCs. First, moDCs were identified based on FSC-SSC parameters excluding debris. The gated moDC population was then further analyzed using contour blot scheme. Isotype controls were used to determine positive/negative thresholds. For phenotypic analysis, if 100% of the cell population was within the positive gate, fluorescence intensity values were used for calculation. Otherwise, the percentage of positive cells was determined. For representative presentation, the contour blots were converted into histograms to better illustrate the overlays.
